# Expression variation in connected recombinant populations of *Arabidopsis thaliana *highlights distinct transcriptome architectures

**DOI:** 10.1186/1471-2164-13-117

**Published:** 2012-03-27

**Authors:** Francisco A Cubillos, Jennifer Yansouni, Hamid Khalili, Sandrine Balzergue, Samira Elftieh, Marie-Laure Martin-Magniette, Yann Serrand, Loïc Lepiniec, Sébastien Baud, Bertrand Dubreucq, Jean-Pierre Renou, Christine Camilleri, Olivier Loudet

**Affiliations:** 1 INRA, UMR1318, Institut Jean-Pierre Bourgin, F-78000 Versailles, France; 2 Department of Plant Genomics Research, INRA, UMR1165 INRA-CNRS, URGV, F-91057 Evry, France; 3 Current address: CEA - DSV - IG - GENOSCOPE, Laboratoire d'Informatique Scientifique, 2 rue Gaston Cremieux, F-91057 Evry, France; 4 AgroParisTech, UMR518 INRA Mathematiques et Informatiques Appliquées, Paris, France; 5 Current address: Institut de Recherche en Horticulture, Semences INRA/Agrocampus Ouest/Université d'Angers, F-49071 Beaucouzé, France

**Keywords:** eQTL, Natural variation, Selection, RILs

## Abstract

**Background:**

Expression traits can vary quantitatively between individuals and have a complex inheritance. Identification of the genetics underlying transcript variation can help in the understanding of phenotypic variation due to genetic factors regulating transcript abundance and shed light into divergence patterns. So far, only a limited number of studies have addressed this subject in Arabidopsis, with contrasting results due to dissimilar statistical power. Here, we present the transcriptome architecture in leaf tissue of two RIL sets obtained from a connected-cross design involving 3 commonly used accessions. We also present the transcriptome architecture observed in developing seeds of a third independent cross.

**Results:**

The utilisation of the novel R/eqtl package (which goal is to automatize and extend functions from the R/qtl package) allowed us to map 4,290 and 6,534 eQTLs in the Cvi-0 × Col-0 and Bur-0 × Col-0 recombinant populations respectively. In agreement with previous studies, we observed a larger phenotypic variance explained by eQTLs in linkage with the controlled gene (potentially *cis*-acting), compared to distant loci (acting necessarily indirectly or in *trans*). Distant eQTLs hotspots were essentially not conserved between crosses, but instead, cross-specific. Accounting for confounding factors using a probabilistic approach (VBQTL) increased the mapping resolution and the number of significant associations. Moreover, using local eQTLs obtained from this approach, we detected evidence for a directional allelic effect in genes with related function, where significantly more eQTLs than expected by chance were up-regulated from one of the accessions. Primary experimental data, analysis parameters, eQTL results and visualisation of LOD score curves presented here are stored and accessible through the QTLstore service database http://qtlstore.versailles.inra.fr/.

**Conclusions:**

Our results demonstrate the extensive diversity and moderately conserved eQTL landscape between crosses and validate the utilisation of expression traits to explore for candidates behind phenotypic variation among accessions. Furthermore, this stresses the need for a wider spectrum of diversity to fully understand expression trait variation within a species.

## Background

Most traits vary quantitatively between individuals and are significantly influenced by genetic variation and its interaction with the environment. In general, transcript abundance of a gene can be considered as a quantitative trait since it differs between individuals with respect to genetic factors [1,2]. The utilisation of technologies such as microarray and, lately, next generation sequencing, allowed the simultaneous quantification of thousands of transcripts, shedding light into the genetics behind gene expression variation [3-5]. Comparison in *Arabidopsis thaliana *showed that at least 46% of the genes in the genome are differentially expressed between a pair of accessions [6]. Variation in gene expression levels is highly heritable and specific genomic regions can be mapped, underpinning transcript abundance variation [1,3] and underlying phenotypic diversity, causing for example differential disease susceptibility in humans [7] or variation in metabolite accumulation in plants [8]... Previous studies in experimental segregating populations have proved the polygenic nature of gene expression variation among lines and mapped thousands of expression quantitative trait loci (eQTLs) in different model organisms, e.g.: *Arabidopsis thaliana *[3,9-12], *Saccharomyces cerevisiae *[13,14], and *Caenorhabditis elegans *[15]. No less important is the interaction between genotypes and the environment [16]. For instance, a third of the budding yeast genes showed a significant strain x condition interaction effect, demonstrating the complexity of the control of transcript abundance.

Transcript level differences can originate from *cis- *and/or *trans*-regulatory changes. *Cis*- acting regulations represent polymorphisms in physical linkage to the gene, affecting the transcription in an allele-specific manner. Consequently, *cis*-eQTLs are usually detected as local-eQTLs, being mapped in the vicinity of the gene; whereas *trans*-acting regulations act distantly in a non allele-specific way and can be located anywhere in the genome, most likely detected as distant-eQTL [1,11,17]. Although linkage or association mapping approaches allow the identification of local- and distant-eQTLs, *cis *and *trans *effects should be directly assessed by allele-specific expression assays [18]. In yeast, most expression differences mapped as major *trans*-acting loci, where few modifiers regulate the expression of hundreds of genes [1,14]. Contrasting with these results, experiments in *C. elegans *revealed an opposite pattern, with an over-representation of local-eQTLs compared to distant regulators [15]. Only few similar studies have addressed this subject in *A. thaliana*. Using microarray technology in recombinant inbred lines (RILs) from a cross between accessions Bayreuth (Bay) and Shahdara (Sha), more than 36,000 eQTLs were described using whole rosettes [3]. In contrast, a parallel study using Landsberg (L*er*) and Cape Verde Island (Cvi) accessions as RIL progenitors, detected 9 times less eQTLs than those mapped in the Bay x Sha population [10]. Discrepancies between the two studies were also evident in the ratio between local- and distant-eQTLs, likely due to differences in statistical power and significance thresholds [11]. Different growth conditions, stages and the lack of similar analytical strategies in these studies impairs fine comparisons between crosses, stressing the need for a systematic approach to better understand the role of natural variation in expression traits among Arabidopsis accessions.

The identification of thousands of local eQTLs allows testing for marks of concerted evolution in transcript abundance from genes with related function [19]. Several studies have addressed this issue and demonstrated the presence of signatures of selection associated with expression traits in a number of systems [5,20]. For example, several pathways were identified in a yeast hybrid between two *Saccharomyces *species exhibiting polygenic directional divergence in *cis*-regulatory variants [5]. Similarly, evidence for selection in expression traits involved in processes such as growth, locomotion and memory was detected in two subspecies of *Mus musculus *[20]. Thus far, evidence for a directional allelic effect between Arabidopsis accessions is scarce. Transcriptome analyses for 18 natural accessions determined an overrepresentation of differentially expressed genes within GO categories, involving response to the biotic environment, among others [6].

In order to identify genomic regions underlying transcript variation in *A. thaliana *and signatures of selection between genes with related function, we performed eQTL mapping in two well-established recombinant populations. The analysis was essentially based on a connected-cross design between three accessions [21]: the reference Columbia (Col-0), Cape Verde Island (Cvi-0) and Burren (Bur-0). Expression level variation was monitored in particular for more than 26,166 annotated nuclear genes in 314 RILs and eQTLs were mapped using a novel R/eqtl package, accurately designed for such a study. Here, we show that the eQTL landscape is moderately conserved across populations and many of the eQTLs identified are cross-specific. Moreover, we show that accounting for confounding sources in one of the crosses increases the sensitivity and the power to detect eQTLs, allowing the identification of a potentially coordinated transcriptional evolution in genes with related functions.

## Results

eQTL analyses were performed in three displays: two on young rosette transcripts in Cvi-0 × Col-0 (hereafter 'CviCol') and Bur-0 × Col-0 (hereafter 'BurCol') RIL sets, and one on developing seed transcripts in Bay-0 × Shahdara (hereafter 'BaySha') RIL set. We will henceforth focus on and compare the results obtained for rosette samples, while the BaySha data is briefly presented as Additional file 1.

Transcript abundance can be treated as a highly heritable phenotype affected by natural genetic diversity [1]. In order to understand the genetics underlying gene expression variation among *A. thaliana *accessions, we utilised the CATMA array technology [22], which has the advantage of not being sensitive to punctual polymorphisms (SNPs) between accessions' transcripts thanks to the length of the probes used (Gene-specific Sequence Tags: GSTs). We measured expression levels for almost 35,000 traits (= GSTs) in three week-old rosettes of 314 RILs obtained from a connected-cross set between three parental accessions (Col-0 as the common male parent, Cvi-0, Bur-0 [21]), including 158 RILs from CviCol and 156 RILs from BurCol. Initially, in order to characterise expression divergence between the accessions selected in this study, the number of differentially expressed genes between pairs of accessions was estimated (Cvi versus Col and Bur versus Col). A total of 1,709 differentially-expressed genes between Col-0 and Cvi-0 and 1,083 between Col-0 and Bur-0 (*P *< 5 × 10^-2^; Additional file 2: Table S1) were detected. This cut-off value represents, at least, 1.3 fold expression differences between the two allelic variants. From this differentially expressed set, none of the parents showed a significant excess of up- or down-regulated genes (49.0% and 54.3% of the probes revealed up-regulation in Cvi and in Bur, respectively, relative to Col). About half of the genes (48.3%) showing significant expression contrast between Col-0 and Bur-0 were also found when comparing the other pair (Additional file 3: Figure S1). Interestingly, 92.3% of this overlap was similarly up- or down-regulated in Col-0 relative to the other two accessions, suggesting that many of the polymorphisms contributing to these differential expression patterns may arise from Col-0 or be shared by Cvi-0 and Bur-0.

### Local and distant eQTL distributions are highly divergent between crosses

**Figure 1:**
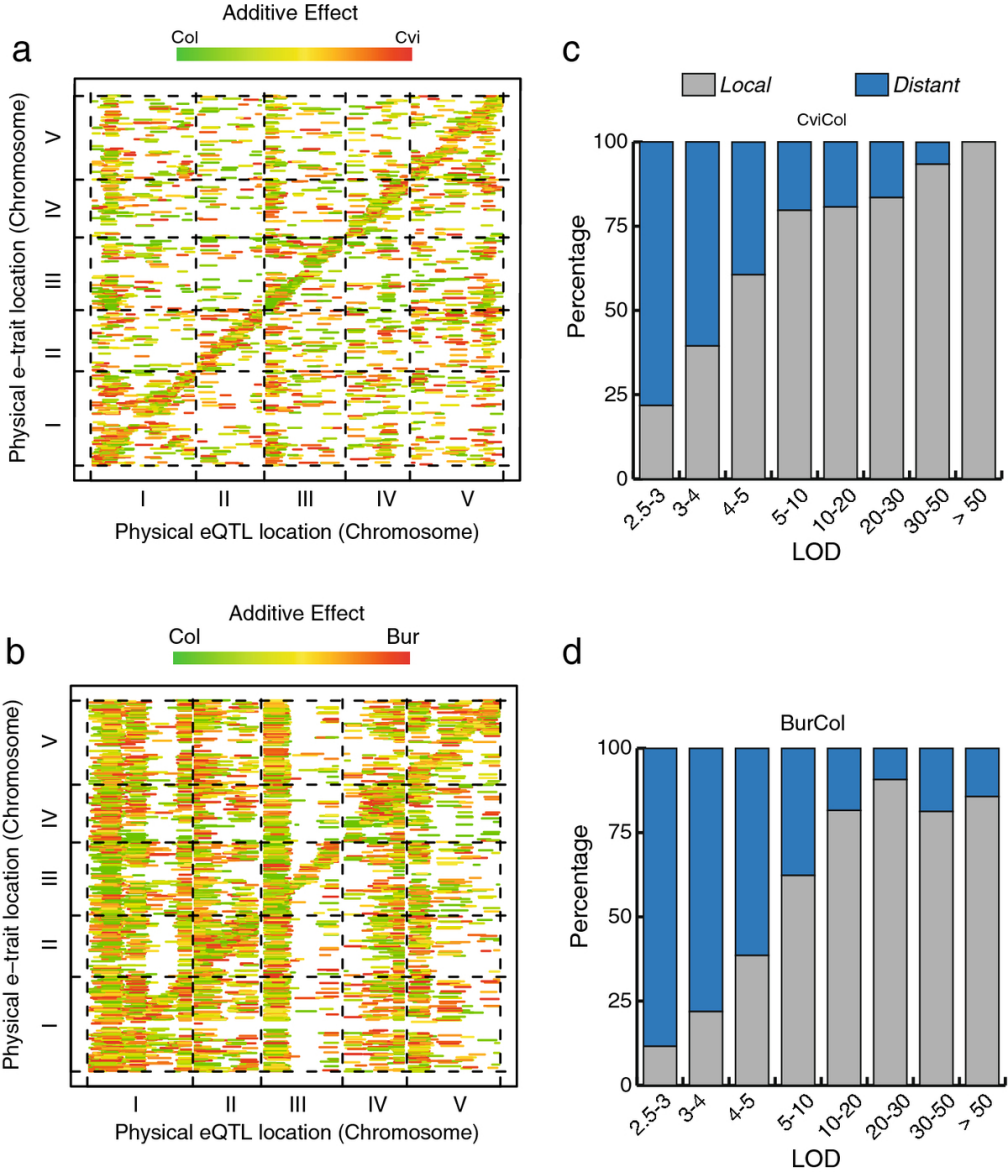
**Genetic landscape for transcript accumulation variation**. **a**. eQTL heatmap for CviCol population significant at a 5% FDR. Each horizontal bar represents an eQTL mapped on the x-axis and controlling the accumulation of a transcript expressed from the locus indicated on the y-axis. The colour of the bar indicates the direction and strength of the eQTL additive effect, and its length along the x axis encompasses the eQTL support interval. Local eQTLs form the diagonal, while distant eQTLs fall elsewhere in the map. **b**. eQTL heatmap for BurCol as described in a. **c**. Bar plot indicating the proportion of local and distant eQTLs for increasing LOD value intervals in CviCol set. **d**. Bar plot as in c. for BurCol set.

In order to detect linkage between genomic regions and transcript accumulation variation, we performed eQTL mapping implementing the novel R/eqtl package (see Methods). We identified 4,290 significant eQTLs at a FDR of 5% (3,906 at 1% FDR) from the CviCol dataset (Figure 1a; Additional file 4: Table S2a) and respectively 6,534 and 5,354 eQTLs from the BurCol dataset (Figure 1b; Additional file 4: Table S2b). This corresponds to a 1:1.5 ratio between sets, at most, a contrast essentially relying on eQTLs of low significance. Among the set of eQTLs in each cross at a FDR of 5%, 2,528 and 4,020 were up-regulated in Col in the CviCol and BurCol populations, respectively. From the set of differentially expressed genes between the parental accessions, 43.1% and 56.9% of them showed a significant linkage to at least one genomic region in the CviCol and BurCol crosses respectively. When comparing the distribution of the phenotypic variance explained per eQTL in both crosses, a highly dissimilar pattern was observed (Kolmogorov-Smirnov test, *P *< 2.2 × 10^-16^). In the CviCol set we found a higher average explained variance (13.3% *vs*. 10.2% in BurCol) with proportionally more eQTLs having larger effects (Additional file 3: Figure S2). Most of the traits were associated with a unique genomic region (89.9% CviCol, 86.3% BurCol), and only a minority exhibited a significant association with two to five eQTLs (Additional file 3: Figure S3). When only considering genes located in the nuclear genome, 13.2% (CviCol) and 19% (BurCol) of these had at least one significant eQTL at a FDR of 5%. Altogether, these results demonstrate the complex nature of transcript variation between accessions and suggest a distinct eQTL landscape for each cross.

Linkage analysis allows the examination of local and distant eQTLs influencing transcript abundance [1,14]. To detect local associations, we set a 1Mb cut-off distance between the eQTL peak and the CATMA GST physical position. Any eQTL reaching a significance peak within 1Mb from the location of the probe it controls was considered as potentially acting in *cis *and hence classified as a local eQTL. In both populations we observed that the distributions of local and distant eQTLs were not uniform throughout the LOD scale (Figure 1c-d). Distant eQTLs were enriched at lower LODs, compared to local eQTLs, which were over-represented at high significance values. Hence, 50% (25.5%) of all eQTLs detected at 5% FDR mapped locally in the CviCol (BurCol) population, while this number would rise to 69.6% (53.6%) if using a much more conservative FDR of 0.1% (*P *< 1 × 10^-4 ^in CviCol and *P *< 5 × 10^-5 ^in BurCol). Moreover, compared to distant eQTLs, local eQTLs explained a significantly higher fraction of the phenotypic variance per trait (Kolmogorov-Smirnov test, *P *< 2.2 × 10^-16^; Additional file 4: Table S2). These results can lead to contrasting conclusions as we vary threshold values (Figure 1c-d). The higher phenotypic variance explained by local eQTLs underlies the fact that most of the phenotypic variation in expression levels is due to local associations, and *trans*-acting factors would have essentially minor effects on single transcripts, but overall a widespread effect over many genes.

**Figure 2:**
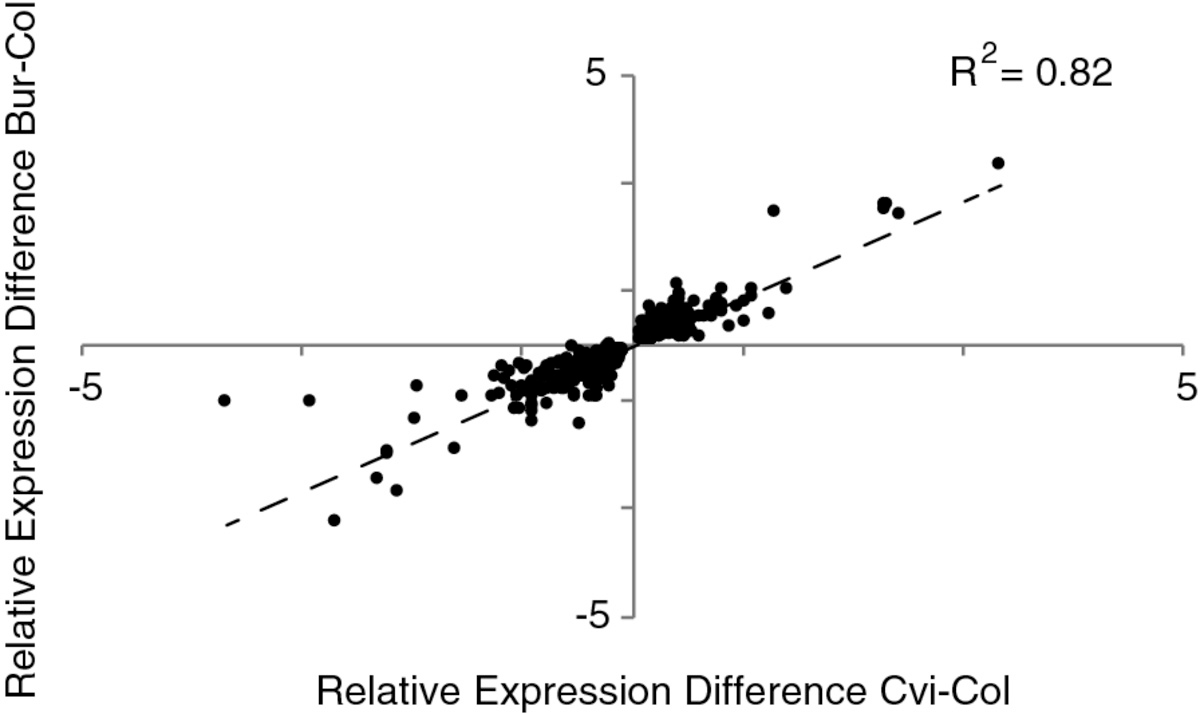
**Correlation between relative expression levels for potentially shared local eQTLs between the CviCol and BurCol sets**. The relative allelic expression level at the eQTL was plotted for transcripts sharing local eQTLs with additive effects in the same direction in both populations. The linear regression and correlation is indicated with a dashed line.

In order to classify cross-specific and overlapping eQTLs, we estimated the number of local eQTLs shared between the BurCol and CviCol populations. We found that 564 transcripts mapped to an overlapping local eQTL interval in the populations, corresponding to 26.3% of all local eQTLs in the CviCol set (Additional file 3: Figure S4). The direction of the allelic effect for these potentially shared eQTLs was consistent in 88.4% of the cases. Moreover, within this subset, we observed a strong correlation between populations for the strength of the additive effect associated with the eQTLs (*R^2 ^*= 0.82; Figure 2) and for the explained phenotypic variance (Spearman correlation test, *P *< 0.01). We also compared the distribution of local eQTLs along the genome between crosses. For this, we divided the genome into 1Mb windows (bin) and estimated the fraction of transcripts with local eQTLs on every bin per chromosome. A spearman test detected a significant correlation between both distributions (*P *< 0.01), suggesting major structural genomic aspects. We determined whether dense eQTL regions were enriched for SNP polymorphisms or were gene-dense regions. A genome-wide regression analysis did not detect a significant correlation between the number of SNPs and eQTLs within intervals for CviCol, however a marginally significant association was found for BurCol (*P *= 0.15 in CviCol, *P *= 0.07 in BurCol). In contrast, an expected significant correlation was found with gene density in both cases (*P *< 2 × 10^-13 ^in CviCol, *P *< 1.3 × 10^-8 ^in BurCol). Furthermore, we compared the top 11 bins (from a total of 116, corresponding to the top 10%) in both sets and detected 4 overlapping intervals with high eQTL density between crosses (Additional file 5: Table S3a). Interestingly, the 4 intervals were not among the top polymorphic or gene-dense and therefore the overlapping cannot be solely attributed to these factors.

### Distant eQTL hotspots are cross-specific

**Figure 3:**
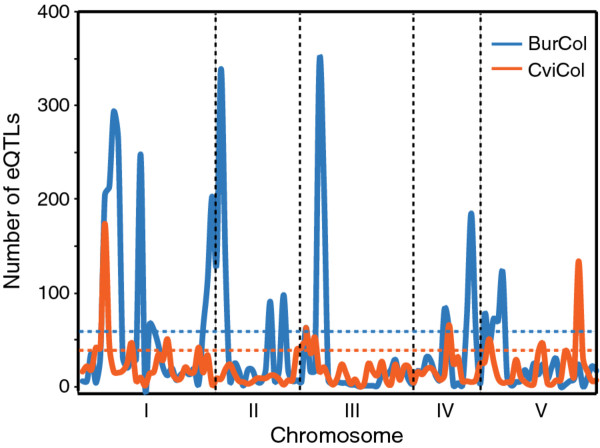
**Distribution of distant eQTLs across the genome and detection of hotspots**. The number of distant eQTLs (y-axis) is plotted against the physical position of the 1Mb-window where they peak (x-axis). In each cross, intervals with an excess of eQTLs relative to the threshold estimated by permutation (dashed lines) were classified as hotspots. This figure refers to Additional file 5: Table S3b.

We considered the set of distant eQTLs mapped at 5% FDR to test for the presence of hotspots along the genome. Similarly to the local eQTL analysis, we divided the genome into 1Mb windows and identified regions containing a higher number of eQTLs than expected by chance, according to a permutation test (see Methods). In the CviCol population, a total of 17 bins contained more than the 34 distant eQTL peaks expected by chance, and up to 170 peaks were found within the strongest hotspot (Figure 3; Additional file 5: Table S3b). These regions included 44.2% of all the distant eQTLs detected. Moreover, on two occasions, two or more adjacent bins were above the threshold, likely highlighting the wide distribution of eQTLs around a major hotspot due to a lack of precision when mapping small-effect loci. For example, the hotspot localised on chromosome 1 included four bins and 13.6% of all the distant-eQTLs, the strongest one in the genome. Within this interval, 170 eQTLs are located at the most dense bin (#6) with an average LOD of 3.6. A different depiction was obtained in the BurCol population: we detected 24 bins containing more eQTLs than expected by chance (> 60 eQTLs; Figure 3). In this population the hotspots encompassed the majority (74.7%) of all the distant-eQTLs. Moreover, within the three major hotspots containing several significant adjacent bins on chromosome 1, 2 and 3 (bins #6-9, #29-31 and #52-53 respectively), we found a significant enrichment for distally-regulated genes of 4 GO categories in the branch 'biological process'. These categories included response to chitin (*P *< 2 × 10^-5^) on chromosome 1 and cell-adhesion (*P *< 1.3 × 10^-5^) on chromosome 2 (Additional file 6: Table S4).

We found little overlap between hotspots in the two populations (Spearman correlation test *P *= 0.21). From the 41 hotspots detected in total, only three were collinear between sets, suggesting little conservation in the major determinants of distant eQTLs across the genome. None of the hotspots co-localised with any of the major-effect developmental QTLs known to segregate in *A. thaliana *(i.e. *ERECTA*, *FRIGIDA*, *CRY2, ..*.), except in BurCol where a marginally significant hotspot was found on chromosome 5 (bin #93), which contains *FLC*. Although 62 traits with a distant eQTL mapping to this region were observed, only 2 have been shown to interact with *FLC *[23], suggesting potentially a minor contribution from this locus. The gene-poor centromeres rich in transposons and pseudogenes were not detected in any of the hotspot intervals. The lack of a significant count of eQTLs extended for at least 1Mb from the centromere until the nearest hotspot, except in the BurCol set where we detected distant eQTLs-rich regions immediately downstream and upstream the centromeres on chromosome 1 and 2, respectively (Figure 3).

### Controlling for confounding factors increases eQTL detection in the CviCol dataset

**Figure 4:**
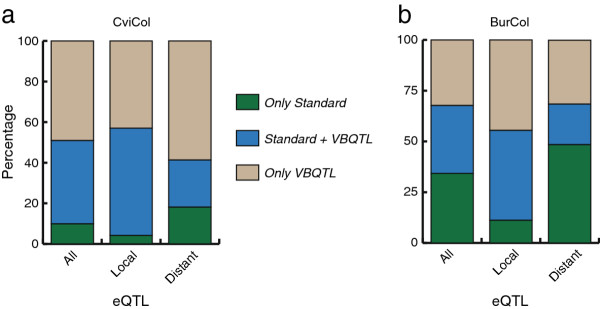
**Bar plot showing overlap and specific eQTL detections when comparing the VBQTL and standard approach**. Linkage mapping methods were compared and blue regions denote the percentage of common eQTLs mapped at a 5% FDR using both approaches. eQTLs solely mapped in one or the other strategy are depicted at the bar-extremes (in green and brown). Ten factors were included in the VBQTL analysis. **a**. CviCol set, **b**. BurCol set.

Variation in transcript abundance can also be influenced by non-genetic factors, such as experimental noise and hidden factors [24,25]: greenhouse localisation, temperature variation, sampling time and other undetected experimental perturbations can affect expression levels among samples, altering the subsequent analysis. A recent association mapping study in *A. thaliana *already demonstrated the greater resolution obtained when considering confounding sources between genotypes (*i.e*. population structure) compared with standard approaches [26]. Hence, it is interesting to try and control these variables in order to improve the eQTL mapping. For this purpose, we utilised the probabilistic approach VBQTL together with R/eqtl to account for hidden confounding factors [24]. We tested this approach in the CviCol dataset for 0, 5, 10, 20 and 30 hidden factors, finding the lowest number of eQTLs for no-hidden factors and the highest for 10 factors (Additional file 3: Figure S5). We chose one known factor (time of harvest) and ten unknown factors to model the expression trait, since the lowest number of distant associations were explained away, in contrast to 20 and 30 factors. Contrarily, using the same number of factors (or less) in the BurCol dataset, we found that many of the distant associations were lost (Figure 4). Using 10 factors we mapped 6,270 eQTLs at 5% FDR (42.6% of them are local) in the BurCol population, while only 29.2% of the distant associations were retained by both the standard and the VBQTL methods. Many of the distant associations exhibiting high significance values were present in both datasets; however, several distant associations with marginally significant LODs were lost (data not shown). The majority of the local associations detected by the standard approach were also mapped by VBQTL (80%). Even more, we observed a 60% increase in the number of local eQTLs detected by VBQTL. Nevertheless, the large number of genetic associations explained away in the BurCol set hindered the subsequent data analysis, since many distant eQTLs affecting transcript levels should be considered (for example, it could be a major regulator causing a hotspot) and not treated as confounding sources [24,27].

We focused the utilisation of this approach on the CviCol population. Linkage analysis detected a total of 8,156 eQTLs (5% FDR; 7,817 at 1% FDR; Additional file 7: Table S5), which is almost twice as many eQTLs as with the standard method, demonstrating the sensitivity of the approach. Interestingly, the fraction of local and distant eQTLs remained almost unchanged. At the less conservative threshold (5% FDR) we mapped 44.6% of the eQTLs in the vicinity of the transcript, slightly less than from the analysis of the raw expression values. Moreover, the contribution to the total number of local and distant eQTLs was increased by 1,461 and 1,025 eQTLs respectively. Many of the local associations mapped by the standard approach were also mapped by VBQTL (92.6%) and, contrarily to the BurCol data set, 56.2% of the distant associations were also retained (Figure 4). Approximately one third of the peaks that had remained only marginally significant (= significant at a lower threshold of 10% FDR) in the standard analysis, were now detected as significant at 5% FDR after VBQTL analysis, likely due to the negative effect of confounding sources on power in the original approach.

### Evidence for directional allelic effect in Col-0 versus Cvi-0 accessions

The independence of eQTLs is crucial for the identification of selection on transcript abundance in a group of genes with related functions [5,20]. In order to detect evidence for a directional allelic effect in the CviCol population, we used the larger set of local eQTLs identified by the VBQTL approach. We considered functional categories with an overrepresentation of eQTLs and a significant bias in the number of genes with either Col or Cvi alleles upregulated. To test for categories with more eQTLs than expected by chance (~1 eQTL every 10 genes, see Methods), the gene functional classification assigned by the Gene Ontology Consortium was used [28]. In particular, we focused on the branch 'biological process', since GO categories here contained could give insight into the divergent adaptation events occurred in each accession. We identified 3 significant overrepresented categories (hypergeometric test, *P *< 8 × 10^-4^, Table 1): "Response to stress", "Response to Biotic and Abiotic stimulus" and "Transport". From the 318 GO sets within these categories, we only analysed those containing more than 20 genes (86 sets). Subsequently, we estimated the number of eQTLs within each set either up or down regulated in any accession and test for deviation from the genome-wide 1.6:1 Col:Cvi eQTL ratio using a hypergeometric test [20]. We found 6 sets showing a significant skew (*P *< 0.05; Table 2), all of them part of the response to stress or biotic and abiotic stimulus, including: defense, hypoxia and plant-type hypersensitive response. Interestingly, in all cases most of the members were upregulated in the Col accession (Table 2). These results demonstrate the presence of a systematic directional allelic effect in sets of genes having related functions and validate the utilisation of expression traits to identify potential intraspecific diversification events.

**Table 1:** Overrepresented GO categories and terms among CviCol local-eQTLs

Functional Category	Expected Number of eQTLs	Observed Number of eQTLs	*P*-value
*cell organization and biogenesis*	165	187	0.034

*developmental processes*	296	283	0.76

*DNA or RNA metabolism*	50	54	0.29

*electron transport or energy pathways*	38	45	0.098

*other biological processes*	274	295	0.074

*other cellular processes*	1457	1534	0.004

*other metabolic processes*	1322	1364	0.041

*protein metabolism*	524	490	0.94

*response to abiotic or biotic stimulus*	276	378	7.70 × 10^-11 ^(S*)

*response to stress*	305	414	2.19 × 10^-11 ^(S*)

*signal transduction*	160	156	0.61

*transport*	240	289	0.0002 (S*)

*unknown biological processes*	1534	1112	1

Number of eQTLs per categories among GO 'Biological process'. Categories with a significant excess (after Bonferroni, *P *< 8 × 10^-4^) of eQTLs are indicated with 'S*'. The number of expected eQTLs was estimated from a whole-genome scan considering the fraction of genes in each functional category

**Table 2:** Directional allelic effect in CviCol

GO term	Genes/term	eQTLs observed	Col up-regulated alleles	Cvi up-regulated alleles	*P*-value (directional allelic effect)
*defense response*	582	58	50	8	0.00002

*response to far-red light*	42	8	8	0	0.02

*plant-type hypersensitive response*	37	11	10	1	0.03

*response to fungus*	35	7	7	0	0.03

*response to oxidative stress*	231	23	18	5	0.04

*response to hypoxia*	20	6	6	0	0.05

GO terms showing a significant directional allelic effect. The number of genes per term, eQTLs observed and number of up-regulating allele from each parent is shown, as well as the *P *value associated with the allelic bias

## Discussion

We have performed quantitative genetic analyses for genome-wide expression traits in a connected-cross set between three accessions in the model organism *A. thaliana*. Utilising the R/eqtl package in both crosses, we observed a 52.3% difference in the number of significant eQTLs between sets, with the Bur-0 × Col-0 cross being the one with the highest count (Figure 1). Regardless of this difference, the conservative figures of eQTLs obtained in both sets resemble the one previously described in RILs between accessions Ler-0 and Cvi-0 [10], but they significantly differ from a previous study involving Bay-0 and Shahdara accessions, where more than 36,000 eQTLs were detected [3]. In our study, the use of a common approach for both sets allowed us to perform straightforward comparisons between them, drawing conclusions not necessarily affected by differences in statistical power [11]. We observed that 17.4% of all local eQTLs detected were shared between crosses, likely due to polymorphisms solely present in Col-0 or shared by Cvi-0 and Bur-0 (although independent SNPs or some allelic heterogeneity pattern could also lead to this observation). An argument to support this hypothesis is the high level of shared eQTLs with an additive effect in the same direction (88.4%) and of similar strength (Figure 2). However, the low level of overlapping eQTLs demonstrates the greater number of cross-specific eQTLs, possibly due to the great complexity of expression traits [1,11,29] and their high potential for evolution [17]. Our results are consistent with a recent survey in several Arabidopsis accessions that identified high allelic heterogeneity within local regulatory eQTLs [6,12], suggesting the existence of many private polymorphisms between all three accessions extending the eQTL landscape.

Although *cis*-acting regulation among local eQTLs remains to be confirmed, the co-localisation of the eQTL and the controlled gene provides a robust probabilistic way of classifying eQTLs into their mode of action. We observed that, albeit the two crosses shared a common accession, the eQTL distribution among these types is very dissimilar. The BurCol set shows three times more distant than local eQTLs. At the same FDR, the CviCol set has a very different balance, with an equivalent number of both types of eQTLs (50% distant and 50% local, Figure 1c) and a lower number of hotspots (Figure 3) encompassing 44.2% of the *trans *eQTLs. Nevertheless, the distribution pattern of local eQTLs on each chromosome was conserved between crosses, likely due to a strong correlation with gene density and chromosome structural features. Also, we found that local eQTLs were overrepresented at higher significance in all crosses (Figure 1c-d), explaining a greater phenotypic variance per trait compared to distant eQTLs. Our result agrees with another study in Mouse, where many highly significant local-eQTLs and moderately significant *trans*-acting associations were observed [29]. Furthermore, the BurCol recombinant population exhibited a higher number of distant eQTLs, concentrating 75% of them in 24 bins classified as hotspots (Figure 3). One of the most interesting intervals was the hotspot at bin #8, which contained the highest number of genes whose function is related to the regulation of transcription (52 genes), including the strong candidate *GIGANTEA *[30], a gene known to be involved in diverse developmental processes [31]. It is difficult to guess whether a hotspot could be the expression of a locus actually controlling the transcription of many genes, or that of a major (for example, developmental) player that has indirect effects on many genes' expression (as secondary consequences of a strong phenotype for example). The presence of pleiotropic hotspots affecting the expression of many transcripts has already been described in yeast [14], where no more than 200 *trans*-eQTLs explained transcript variation for 1,716 traits. Similarly, many *trans*-hotspots were described in previous *A. thaliana *studies [3,10]. In agreement with these studies, the hotspots detected here explained approximately 10% of the individual traits' variation, demonstrating their milder effect on transcript abundance compared to local eQTLs [11]. Interestingly, only ~7% of the hotspots overlapped between crosses, suggesting either alternative master regulators or little conservation in complex regulatory networks [14]. These conserved regions could also suggest the presence of polymorphisms in Col-0 as major contributors for the large number of *trans *eQTLs within these shared regions.

To increase the power in detecting eQTLs and identify milder genetic variations, we have utilised the bayesian framework VBQTL, a model designed to dissect gene expression variation in order to account for confounding factors [24]. The implementation of this model in the CviCol set allowed us to detect twice as many eQTLs compared to the standard approach (Figure 4), without affecting the ratio of local *versus *distant eQTLs. Subsequently, we used this extended set and focused the posterior analyses on local eQTLs since they likely represent independent alleles, only affecting a single transcript and explaining a greater phenotypic variance, in contrast to distant eQTLs that may affect many genes and show minor phenotypic effects. The use of this new set of local eQTLs allowed us to look for potential traces of selection where Col alleles were overexpressed in a set of genes with related function. Assuming neutrality, no over-representation of either up-regulated or down-regulated alleles from the same accession is expected within a cluster of genes, unless a directional allelic drift has occurred, which may represent selection [32]. We detected an overrepresentation of eQTLs in several GO functional categories and a significant skew within GO terms (Table 1, 2). The accumulation of local regulatory polymorphisms up-regulating Col alleles in all cases, suggests dissimilar patterns of responses to the environment and an advantage in a particular niche [5] or, else, a specific lack of cost in loosing this response. For example, one of the clusters of genes globally up-regulated in Col with respect to Cvi relates to hypoxia response, which is a major determinant of submergence tolerance. It was indeed recently shown that Col and Cvi contrasts for this trait, with Cvi being one of the least submergence-tolerant accessions [33]. Interestingly, the entire set of significant categories here described are related to response to stress or biotic and abiotic response, highlighting its potential link with environmental and niche-adaptation changes.

## Conclusions

Our results demonstrate the importance of extending the number of mapping populations in order to understand the eQTL landscape within a species. We found that the distribution of local and distant eQTLs is moderately conserved between recombinant populations, demonstrating the complex inheritance of transcript abundance. Moreover, the identification of candidate pathways for signatures of selection is a significant step towards understanding accession diversification and their adaptation to the environment. Further studies using novel technologies, such as next generation sequencing (RNA-seq), association mapping, or the combination of both, will help elucidating and validating transcript abundance divergence with a greater resolution within *A. thaliana *species.

## Methods

### Plant material, sample preparation and microarray hybridization

We used 158 RILs from the core-population of the Cvi-0 × Col-0 set and 156 RILs from the core-population of the Bur-0 × Col-0 set [21], along with their respective parents. Plants were cultivated in a greenhouse in typical long day conditions (16h photoperiod) at 20°C and whole plants were collected above the roots 20 days after sowing, which corresponds on average to growth stage '1.08' from Boyes et al. [34]. Total RNA was extracted using RNeasy Plant mini Kit (Qiagen kit #74904) according to the supplier's instructions. For each biological sample (RIL), total RNAs were obtained by pooling RNAs from three randomized plants within a single experiment.

Transcript abundance estimates were carried out using the bicolour CATMA microarrays version 5, containing 34,529 spots (including multiple internal controls) allowing, among others, to uniquely interrogate 26,166 nuclear genes (predicted from TAIR and EUGENE algorithms) in *A. thaliana *[22,35]. For each comparison of two RILs (random pair design), one technical replicate with fluorochrome reversal was performed (i.e. four hybridisations -2 arrays- per comparison of two RILs). Labelling of cRNAs with Cy3-dUTP or Cy5-dUTP (Perkin-Elmer-NEN Life Science Products), hybridisation to the slides, and scanning procedures were performed as previously described [36].

### Microarray data analysis

For each array, the raw data comprised the logarithm of the median feature pixel intensity at wavelengths 635 nm (red) and 532 nm (green) and no background was subtracted. An array-by-array normalisation was performed to remove systematic biases. First, spots considered as badly formed features were excluded. Then a global intensity-dependent normalisation using the loess procedure was performed to correct the dye bias [37]. Finally, for each block, the log-ratio median calculated over the values for the entire block was subtracted from each individual log-ratio value to correct print tip effects. Differential analysis was based on the log ratios averaged on the dye-swap: the technical replicates were averaged to get one log-ratio per biological replicate and these values were used to perform a paired *t*-test. A trimmed variance is calculated from spots which do not display extreme variance [38]. The raw *P*-values were adjusted by the Bonferroni method, which controls the Family Wise Error Rate in order to keep a strong control of the false positives in a multiple-comparison context. We considered probes as being differentially expressed after Bonferroni correction using a *P *< 0.05. For the eQTL analysis, normalised intensity per probe × RIL has been calculated from the normalised log-ratio by sharing the correction value between both samples [39]. To be specific, after the normalization of each array, we get a log-ratio denoted M and a mean intensity denoted A for each probe. First, we average M and A across the two arrays taking into account the dye switching and then calculate the normalised intensity of the two co-hybridized RILs which are equal to (2A-M)/2 and (2A + M)/2. The intensity (2A-M)/2 corresponds to the RIL labelled in green on the first array of the dye-swap. Then a between-array normalisation was performed to rescale the mean intensity of each slide at an arbitrary value equal to 8.5. This second round of normalisation makes probe × RIL intensity comparable and gives us a single expression level for each probe × RIL that can be used further for the eQTL mapping strategy.

### Data Deposition

Microarray data was deposited at Gene Expression Omnibus http://www.ncbi.nlm.nih.gov/geo/, accession no. GSE28791 and at CATdb (http://urgv.evry.inra.fr/CATdb/; Project: GNP07_RILKIT) according to the 'Minimum Information About a Microarray Experiment' standards.

### R/eqtl package and eQTL mapping

eQTL mapping was performed utilising the normalised microarray data using our R package R/eqtl http://cran.r-project.org/web/packages/eqtl/, which exploits functions from R/qtl [40]. R/eqtl is designed specifically for the mapping of thousands of traits in parallel. It includes functions to systematically and automatically identify and select QTLs with supporting intervals from R/qtl simple genome scan results (interval mapping), defining QTL as covariates for a composite interval mapping, calculating additive effect, estimating heritability for single QTL and for QTL × QTL interactions, classifying eQTL as candidate for *trans*- (distant) and *cis*-acting (local) eQTL (according to the position of the controlled gene with respect to the eQTL support interval and/or an arbitrary physical window), projecting and plotting the QTL estimated location relative to the gene location and summarising eQTL data at the genome scale with various generic plots and tabular files. R/eqtl is a free and open-source multi-platform package developed under the statistical language R, and is available under the GPLv3 license. R/qtl and its extension R/eqtl are hosted and can be downloaded from the Comprehensive R Archive Network (CRAN) at http://cran.r-project.org.

Interval Mapping (IM) and Composite Interval Mapping (CIM) were performed on each cross for all of the 32,300 non-technical and non-repetitive probes on the CATMA array, as implemented in the R/eqtl package. The Cvi × Col and Bur × Col RIL populations have been previously genotyped for 90 and 87 markers, respectively [21]. For each probe, IM was first applied to identify a primary set of eQTLs and then this set was used as co-factor in CIM to detect less significant QTLs. Taking into account population size and, hence, the number of observed informative recombinants that conditions the ability to distinguish two linked QTL, a 15 cM exclusionary window on each side of all QTL peaks was applied to refrain from trying to detect too closely linked eQTLs or interpreting large peaks into multiple eQTLs. The analysis was completed with the estimation of the additive effect by averaging the phenotype value for each allele at the QTL marker (or pseudomarker) and estimating the relative contribution of each allele as Xxx *minus *Col (so that a negative allelic effect indicates that Col is up-regulated with respect to Xxx). We also estimated the proportion of the phenotypic variation explained by the segregation of each individual eQTL or significant eQTL × eQTL interaction (R^2^) by analysis of variance. QTLs were defined using a LOD significance threshold computed by permuting the phenotypes while maintaining the genotype across the RIL set [41] and a 1.5 LOD drop-support interval [42]. In order to obtain a genome-wide threshold we determined the 95-percentile permutation threshold among 500 randomly chosen traits and estimated the 95% upper-bound from the traits distribution. We called an eQTL significant if the LOD score was above the threshold (genome-wide False Discovery Rate, FDR = 0.05)[3,16]. To correct for multiple-traits testing, we estimated the minimum *P*-value from each randomly chosen trait and estimated the corresponding q-value [43]. *P*-values for transcriptome-wide FDR 5% (*P *= 6 × 10^-3 ^in CviCol and *P *= 9 × 10^-3 ^in Burcol) and 1% (*P *= 1 × 10^-3 ^in CviCol and BurCol) were also used as significance threshold.

### QTLstore

All results are recorded in QTLstore, a comprehensive web service which allows to store, manage, explore and cross QTL experiment results from the single-trait scan to genome-wide expression QTL experiments. QTLstore is hosted at http://qtlstore.versailles.inra.fr/ and is composed of an extensive SQL database and a web interface, which are available under the GPLv2 license. The SQL database is convenient for all kinds of QTL experiments and analysis, by being able to store all the experiments primary data separately from all subsequent analyses results. It typically stores and interrogates QTL parameters such as location, significance, effect and type, among others.

### Local and distant eQTL distribution

Local and distant eQTLs were classified in 1Mb bins as in [1]. Briefly, we divided the genome into 116 physical bins (independent of the number of markers on each population) of 1Mb each (the chromosome ends were included into the previous 1Mb bin if the remaining region was smaller than 500 Kb). For distant eQTLs we performed a permutation test as previously described [3]. Bins containing a higher number than the maximum expected by chance (α = 0.05) were considered as *trans*-hotspots. For local eQTLs, we performed a regression analysis against SNP (for Cvi-0 and Bur-0, SNP data was downloaded from the 1001genomes project website http://1001genomes.org/) and gene densities as described in [10]. Significance was estimated using a one-way ANOVA. Significance for GO enrichment was assessed utilising a *P *< 2 × 10^-5 ^after Bonferroni correction.

### VBQTL and test for directional allelic effect

We applied the VBQTL (Variational Bayesian QTL Mapper) approach as previously described [24] on the dataset obtained in the Cvi-0 × Col-0 population, using one known factor to model hidden confounding sources. Residuals of the estimated effect were used as phenotypic values and eQTLs were detected again using R/eqtl and the statistical procedure implemented for the standard approach (*P *< 1 × 10^-2^, FDR 5%). We tested for 5, 10, 20 and 30 factors and chose the model with the highest number of additional linkages (FDR 5%). Next, potential directional selection was tested as previously described with modifications [20]. Briefly, only local eQTLs were retained after the VBQTL analysis and tabulated based on their gene ontology (GO) classification. Initially, we selected functional categories with a significant over-representation of eQTLs within the 'Biological process' set. For this purpose, we assessed significance utilising the hypergeometric test (*P *< 8 × 10^-4 ^after Bonferroni) considering the number of genes within each category at the whole-genome level. Within the selected functional categories, we focused the subsequent analysis on GO categories containing more than 20 genes. For each category we tested departure from the 1.6:1 ratio (Col:Cvi) using a hypergeometric test.

## Competing interests

The authors declare that they have no competing interests.

## Authors' contributions

OL, CC, JPR, LL, SeB and BD designed the study. JY, CC, SeB, BD, SaB and SE handled plant culture for RNA. JY, SE, SaB and MLMM performed the array experiments and initial data analyses. HK, JY, FAC, MLMM and OL developped R/eqtl and analysed the statistical data. HK and YS developped databases and web tools. FAC and OL wrote the manuscript, while JY, HK, MLMM, LL, SeB, BD, JPR and CC corrected the manuscript. All authors read and approved the final manuscript.

## Supplementary Material

Additional file 1 **eQTL mapping in the Bay × Sha recombinant population **[44]. Click here for file

Additional file 2 **Table S1**. Set of differentially expressed genes between parental accessions Cvi, Bur and Col. Expression differences are reported in log_2_. Click here for file

Additional file 3 **Figure S1**. Venn diagram depicting the overlap between genes with differential expression in parental accessions pairs (Cvi *vs*. Col and Bur *vs*. Col). **Figure S2**. Histograms of the explained phenotypic variance (R2; %) for the eQTLs in the a.CviCol and b. BurCol populations. **Figure S3**. Number of eQTLs per trait. **Figure S4**. Venn diagram depicting the overlap between probes with local eQTLs in the CviCol and BurCol populations. **Figure S5**. Histogram of the number of probes with a significant eQTL for different numbers of hidden factors tested with VBQTL in CviCol. **Figure S6**. Genetic landscape for transcript accumulation variation in BaySha. **Figure S7**. Histogram of the explained phenotypic variance (R2) for the eQTLs in the BaySha population. **Figure S8**. Number of eQTLs per trait in BaySha. **Figure S9**. Distribution of distant-eQTLs along the genome in BaySha. Click here for file

Additional file 4 **Table S2**. List of eQTLs detected in each cross. Abbreviations: eQTL Chr = chromosome localisation of eQTL, type = eQTL tentative classification ('cis'/'trans' = local/distant), peak.bp = physical position of the LOD peak, inf.pb = inferior limit, sup.pb = superior limit of supporting physical interval, Add = additive effect (estimated as Xxx-Col, so that a negative allelic effect means Col up-regulated with respect to Xxx), Rsq = variance explained, Rpf = significance. Click here for file

Additional file 5 **Table S3**. Number of -eQTLs per 1 Mb interval. a. local, b. distant. Click here for file

Additional file 6 **Table S4**. Overrepresented GO terms within the BurCol distant-hotspots. Click here for file

Additional file 7 **Table S5**. List of eQTLs detected by VBQTL in CviCol. See Additional file 4: Table S2 for column headings. Click here for file
