# Supplementary material for: Expression variation in connected recombinant populations of Arabidopsis thaliana highlights distinct transcriptome architectures
Source: BMC Genomics. 2012 Mar 27;13:117. doi: 10.1186/1471-2164-13-117 (PMC3359214; doi:10.1186/1471-2164-13-117)
Supplement: Additional file 1 — eQTL mapping in the Bay × Sha recombinant population [44]. [file 1471-2164-13-117-S1.PDF]

Supplementary Text

eQTL mapping in the Bay x Sha recombinant population.

Expression levels for 24,576 traits (Gene-specific Sequence Tags: GSTs, CATMA array version 2) was measured in RNA extracted from developing seeds (harvested 10 days after pollination) of 157 RILs from the Bay-0 x Sha population (hereafter BaySha)[1]. Display, sample treatment, microarray analysis and data normalisation were performed as indicated in Methods within the main text. We used this dataset to develop and test the efficiency of the novel R/eqtl package as a tool for identifying expression quantitative trait loci (eQTLs) in recombinant populations.

Linkage analyses on expression traits allowed us to identify 19,021 significant eQTLs (*FDR* = 5%, LOD > 2.47 after 1,000 permutation tests on 100 randomly chosen expression traits; **Figure S6a; Table S2c**). For each of them, the variance explained, the supporting interval, the additive effect and the classification as potentially *cis*- or *trans*-acting eQTL (see main text for details) were determined. The accurate results provided by R/eqtl in the BaySha population allowed us to perform further mapping and crosses comparisons with the microarray data generated in the CviCol and BurCol recombinant populations.

The number of eQTLs identified in BaySha exceeds those ones found for the CviCol and BurCol recombinant populations by 4.4 and 2.9 fold, respectively. The majority of the eQTLs were mapped as distant (91.7%), and only a minority (8.3%) was found to act locally (**Figure S6a-b**). The significant over-representation of distant association and the total lower number of local-eQTLs detected in the BaySha population explains the greater difference between this set and the two crosses involving the Col-0 accession. Nevertheless, in agreement with CviCol and BurCol findings, we observed a higher average variance explained by local eQTLs compared to distant-eQTLs (13.2% *vs.* 11.3% respectively, $P < 2.2e-16$, **Figure S6b, Figure S7**) with more local-eQTLs having larger effects and significance (**Figure S6b**). Most of the traits were associated with a unique genomic region (**Figure S8**), in agreement with CviCol and BurCol findings.

The distribution of the distant-eQTLs along the genome was not uniform and most of them gathered in specific regions of the genome (**Figure S9**). In order to map intervals significantly over-represented with distant-eQTLs (hotspots), we performed a permutation test after dividing the genome into 1Mb intervals (see Methods in main text). Only three significant hotspots were detected localised in the left arm of chromosome 4 and 5. Interestingly, two well-characterised pleiotropic QTLs known to segregate in this cross were found within these regions. Below the strongest hotspot (gathering ~12,000 eQTLs on chromosome 4) lies the flowering time gene *FRIGIDA* (*FRI*) as an obvious effector. Similarly, the second major hotspot, linked to the expression of over 2,000 genes, localised on chromosome 5 in an

interval neighbouring the *FLC* locus. Variation at *FRI* and *FLC* is known to control most of the variation for flowering time in this cross [1]. Additionally, these hotpots are not independent, with most of the genes controlled by a distant-eQTL at *FLC* also controlled by a distant-eQTL at *FRI*, in most instances with opposite allelic effects. In other words, this means that hundreds of transcripts have a genetic architecture (= detected QTLs and their parameters) which is very close to the one obtained for the flowering precocity trait itself. Although *FLC* has been hypothesised to directly regulate many genes outside of the flowering time pathway [2], the observed pattern fits better with an indirect effect of flowering time on the timing of gene expression in the developing seeds after pollination.

Previously, linkage mapping of rosette transcripts for this line detected 36,871 eQTLs, which is almost twice as much as detected in this study. Contrary to our results, many of the eQTLs gathered in a distant-hotspot localised on chromosome 2 and no evidence for a large number of transcripts regulated by either *FRI* or *FLC* was reported [3]. Discrepancies between studies could be explained by the different stages at which very different tissues were sampled, where different *trans*-factors could lead the expression of thousands of transcripts.

The BaySha developping seeds eQTLs results are stored in the QTLStore portal.

References

1. Loudet O, Chaillou S, Camilleri C, Bouchez D, Daniel-Vedele F: **Bay-0 x Shahdara recombinant inbred line population: a powerful tool for the genetic dissection of complex traits in Arabidopsis**. *Theor Appl Genet* 2002, **104**:1173-1184.
2. Deng W, Ying H, Helliwell CA, Taylor JM, Peacock WJ, Dennis ES: **FLOWERING LOCUS C (FLC) regulates development pathways throughout the life cycle of Arabidopsis**. *Proc Natl Acad Sci USA* 2011, **108**(16):6680-6685.
3. West MA, Kim K, Kliebenstein DJ, van Leeuwen H, Michelmore RW, Doerge RW, St Clair DA: **Global eQTL mapping reveals the complex genetic architecture of transcript-level variation in Arabidopsis**. *Genetics* 2007, **175**(3):1441-1450.
