# Supplementary material for: Expression variation in connected recombinant populations of Arabidopsis thaliana highlights distinct transcriptome architectures
Source: BMC Genomics. 2012 Mar 27;13:117. doi: 10.1186/1471-2164-13-117 (PMC3359214; doi:10.1186/1471-2164-13-117)
Supplement: Additional file 3 — Figure S1. Venn diagram depicting the overlap between genes with differential expression in parental accessions pairs (Cvi vs. Col and Bur vs. Col). Figure S2. Histograms of the explained phenotypic variance (R2; %) for the eQTLs in the a.CviCol and b. BurCol populations. Figure S3. Number of eQTLs per trait. Figure S4. Venn diagram depicting the overlap between probes with local eQTLs in the CviCol and BurCol populations. Figure S5. Histogram of the number of probes with a significant eQTL for different numbers of hidden factors tested with VBQTL in CviCol. Figure S6. Genetic landscape for transcript accumulation variation in BaySha. Figure S7. Histogram of the explained phenotypic variance (R2) for the eQTLs in the BaySha population. Figure S8. Number of eQTLs per trait in BaySha. Figure S9. Distribution of distant-eQTLs along the genome in BaySha. [file 1471-2164-13-117-S3.PDF]

Supporting Figures

Figure S1. Venn diagram depicting the overlap between genes with differential expression in parental accessions pairs (Cvi *vs.* Col and Bur *vs*. Col).

Figure S2. Histograms of the explained phenotypic variance (R^2; %) for the eQTLs in the a.CviCol and b. BurCol populations.

Figure S3. Number of eQTLs per trait. The percentage and number of traits explained by 1 to 5 eQTLs are indicated along the y-axis and on top of each bar, respectively. **a**. CviCol **b**. BurCol at a FDR of 5%.

Figure S4. Venn diagram depicting the overlap between probes with local eQTLs in the CviCol and BurCol populations.

Figure S5. **Histogram of the number of probes with a significant eQTL for different numbers of hidden factors tested with VBQTL in CviCol.**

Figure S6. Genetic landscape for transcript accumulation variation in BaySha. a. eQTL heatmap for BaySha population significant at a 5% FDR. Each horizontal bar represents an eQTL mapped on the x-axis and controlling the accumulation of a transcript expressed from the locus indicated on the y-axis. The colour of the bar indicates the direction and strength of the eQTL additive effect, and its length along the x axis encompasses the eQTL support interval. Local eQTLs form the diagonal, while distant eQTLs fall elsewhere in the map. **b.** Bar plot indicating the proportion of local and distant eQTLs for increasing LOD value intervals.

Figure S7. Histogram of the explained phenotypic variance (R^2) for the eQTLs in the BaySha population

Figure S8. Number of eQTLs per trait in BaySha. The percentage and number of traits explained by 1 to 5 eQTLs are indicated along the y-axis and on top of each bar, respectively.

Figure S9. Distribution of distant-eQTLs along the genome in BaySha. The number of eQTLs (y-axis) is plotted against the physical position of the 1Mb-window where they peak (x-axis). Intervals with an excess of eQTLs relative to the threshold estimated by permutation (red dashed line) were classified as hotspots.

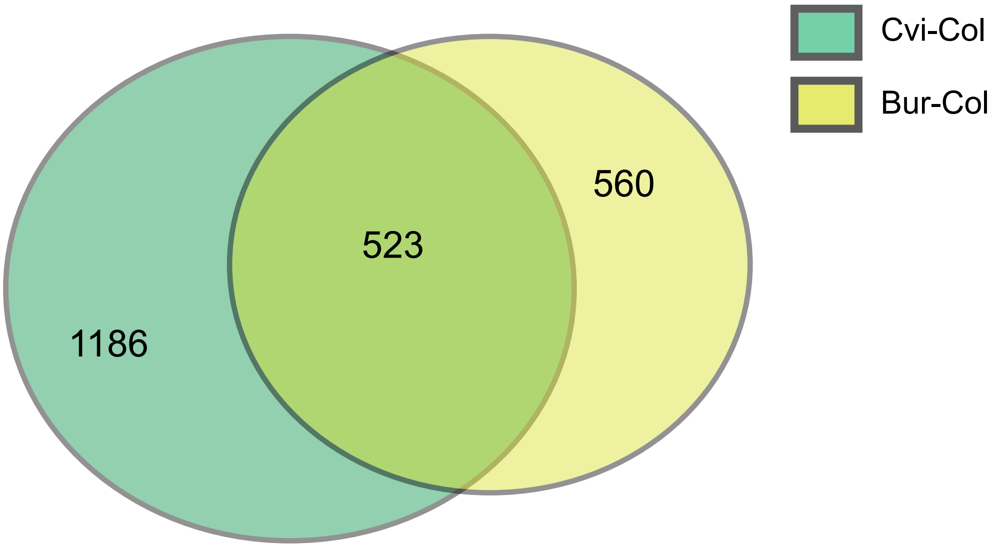

Figure S1

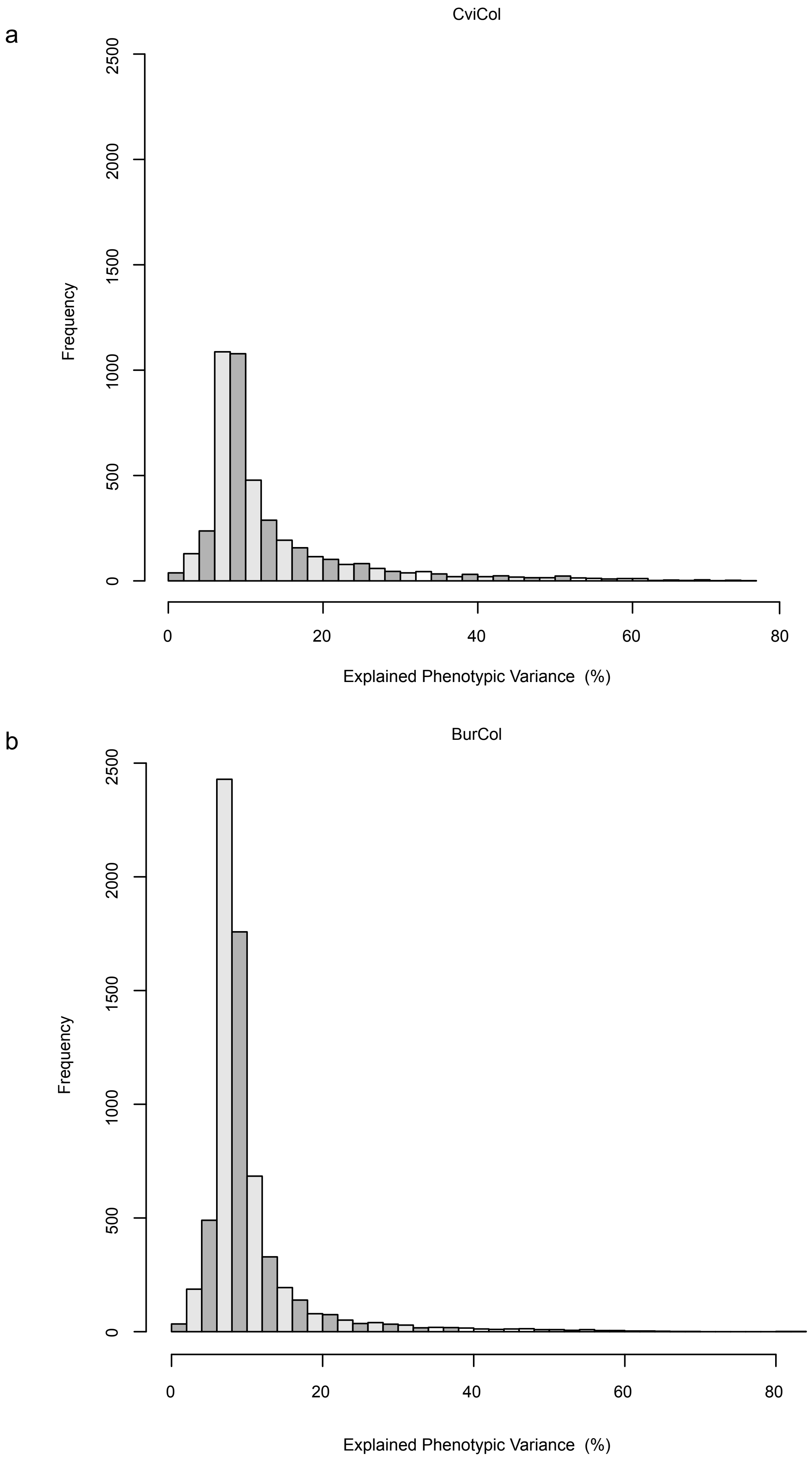
a
CviCol
Frequency
0
500
1000
1500
2000
2500
0
20
40
60
80
Explained Phenotypic Variance (%)
b
BurCol
Frequency
0
500
1000
1500
2000
2500
0
20
40
60
80
Explained Phenotypic Variance (%)

Figure S2

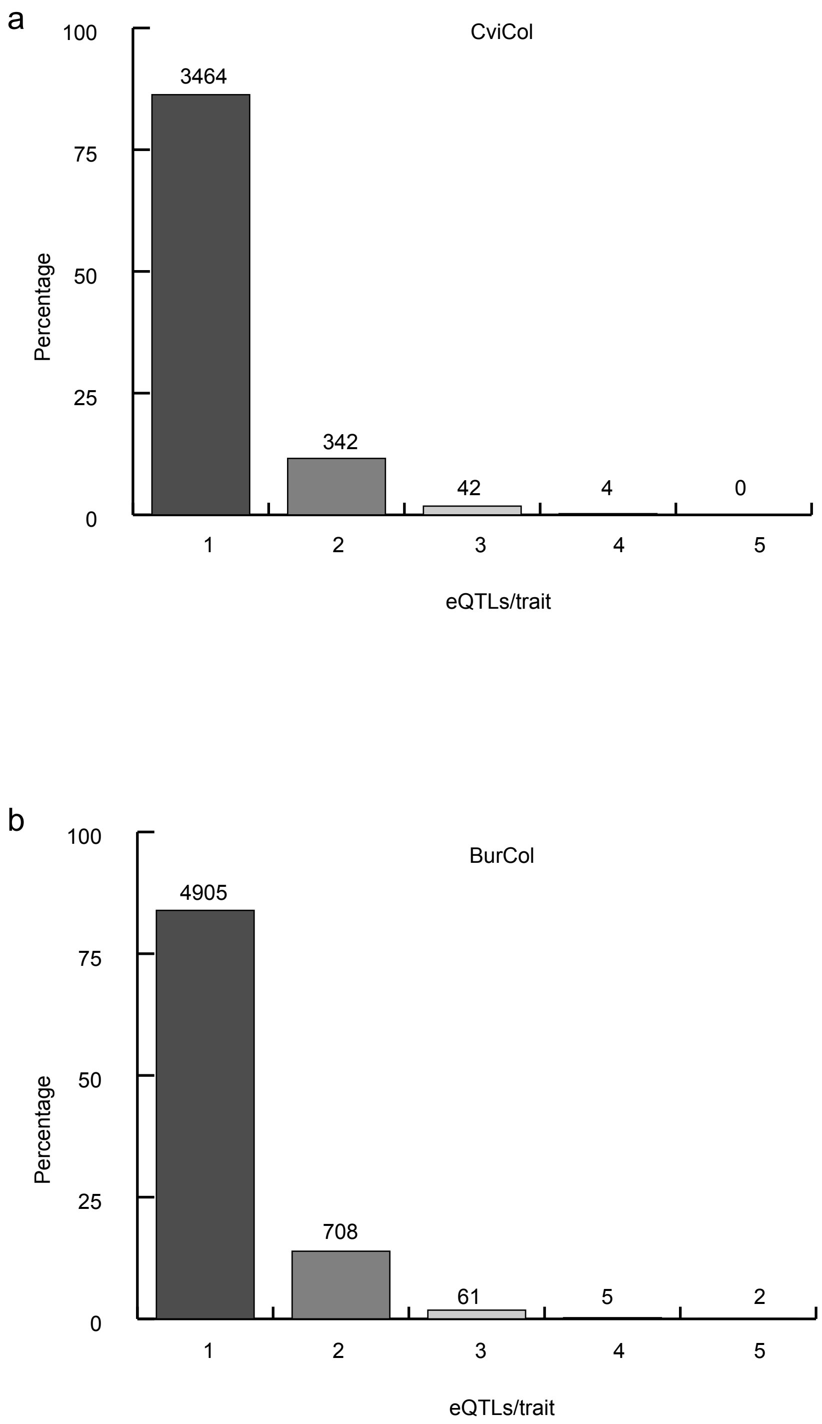
a
CviCol
100
75
50
25
0
Percentage
3464
342
42
4
0
1
2
3
4
5
eQTLs/trait
b
BurCol
100
75
50
25
0
Percentage
4905
708
61
5
2
1
2
3
4
5
eQTLs/trait

Figure S3

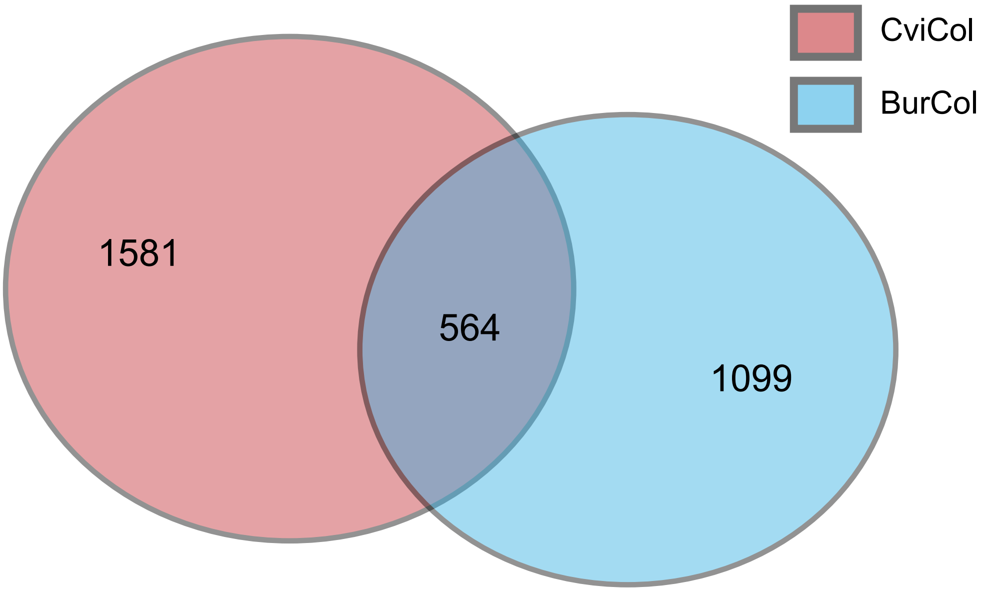

Figure S4

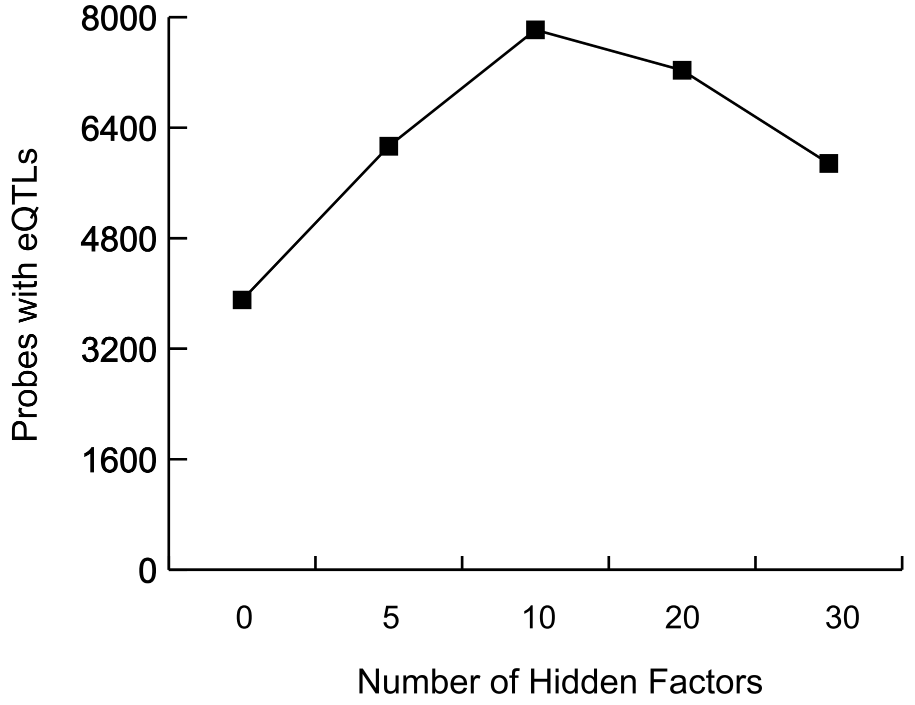
8000
6400
4800
3200
1600
0
Probes with eQTLs
0
5
10
20
30
Number of Hidden Factors

Figure S5

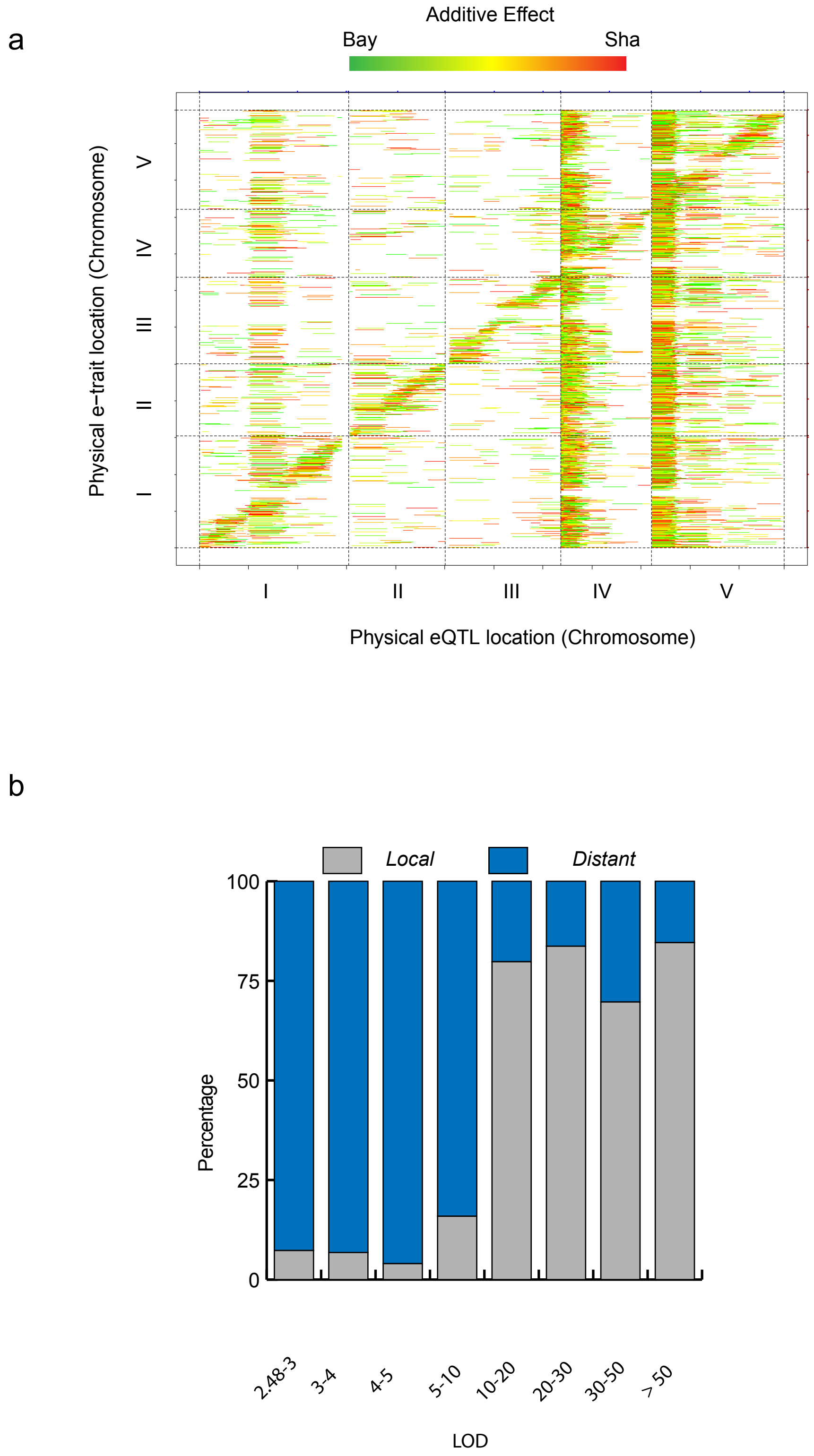
a
Additive Effect
Bay
Sha
Physical e-trait location (Chromosome)
V
IV
III
II
I
I
II
III
IV
V
Physical eQTL location (Chromosome)
b
Local
Distant
100
75
50
25
0
Percentage
2.48-3
3-4
4-5
5-10
10-20
20-30
30-50
> 50
LOD

Figure S6

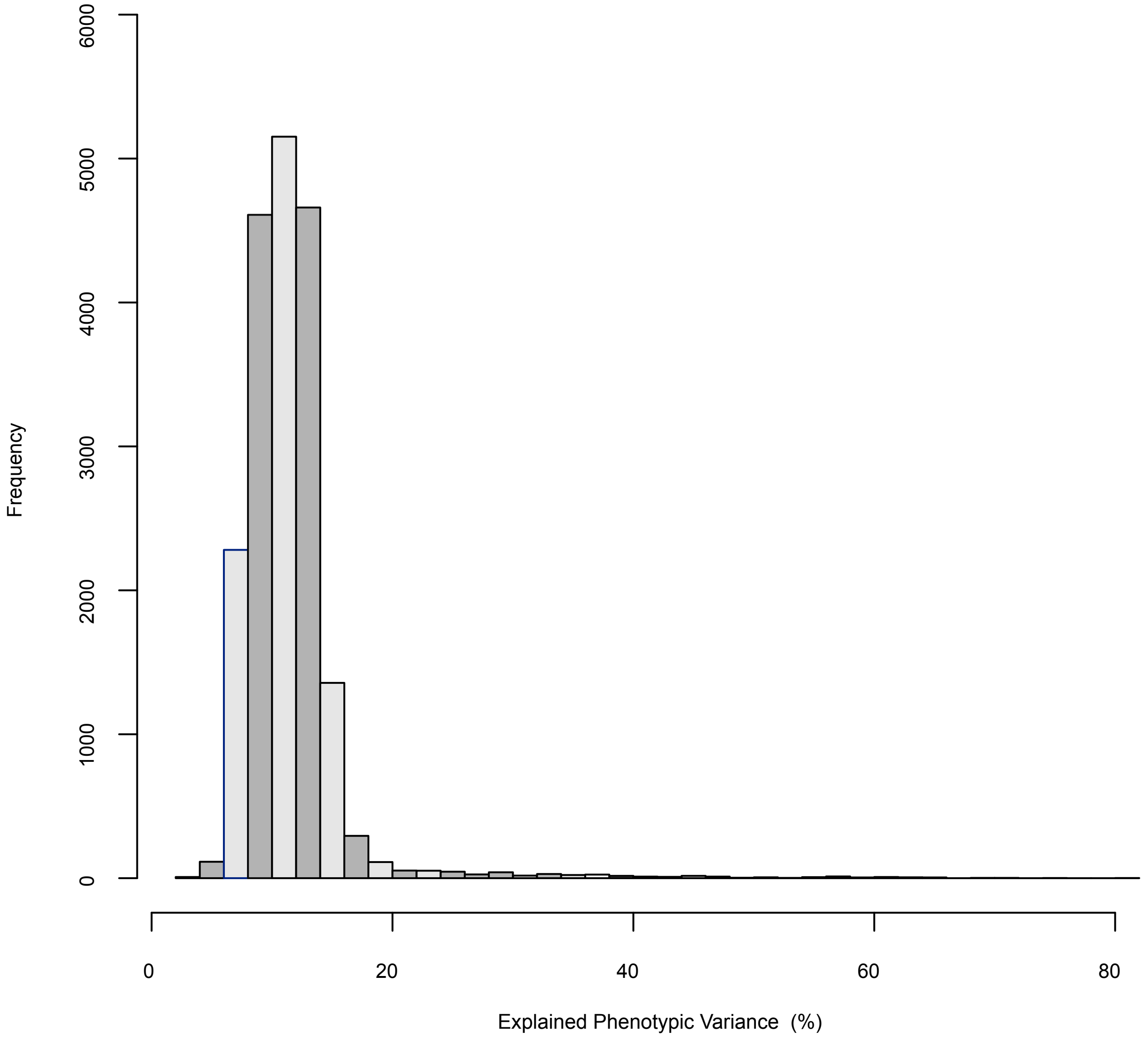

Frequency
0
1000
2000
3000
4000
5000
6000
0
20
40
60
80
Explained Phenotypic Variance (%)

Figure S7

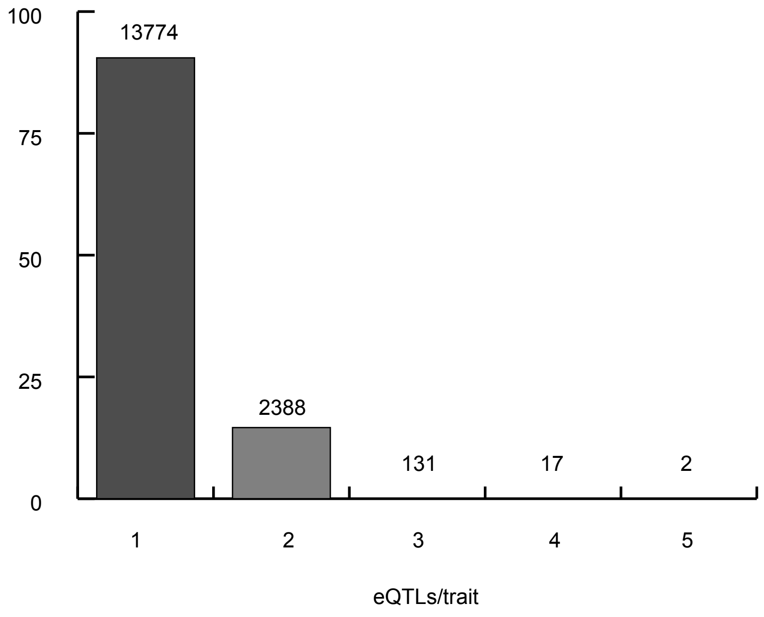

Figure S8

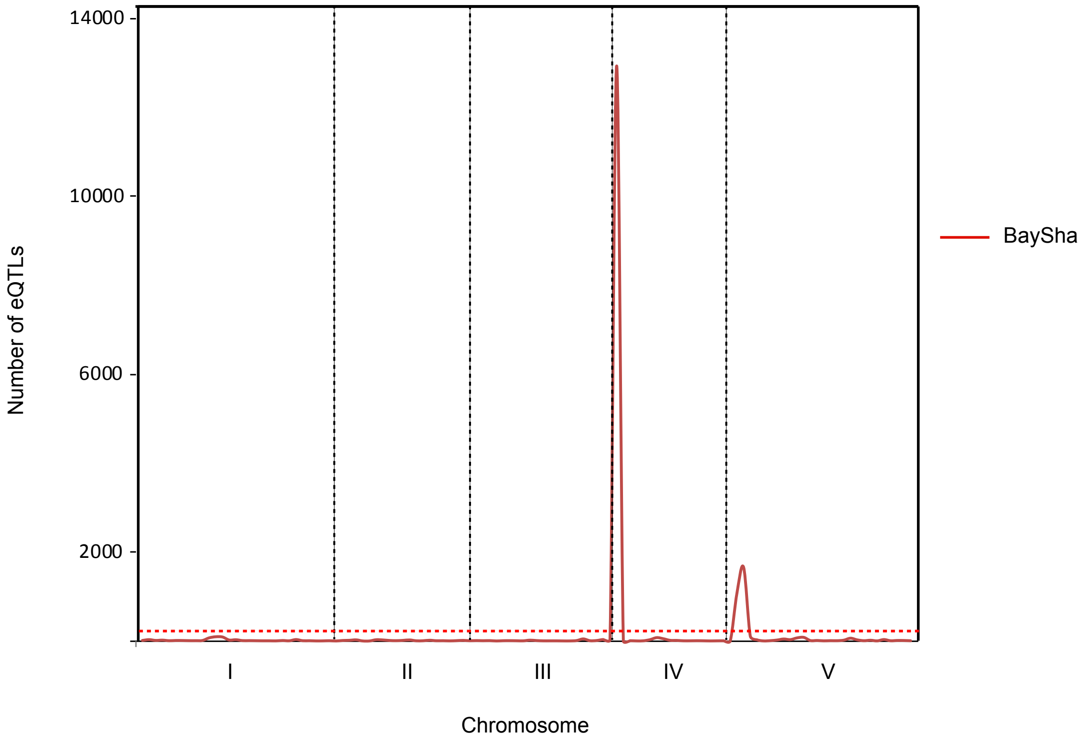
14000
10000
6000
2000
Number of eQTLs
I
II
III
IV
V
Chromosome
BaySha

Figure S9
